# Three‐dimensional bladder ultrasound for estimation of urine volume in dogs compared with traditional 2‐dimensional ultrasound methods

**DOI:** 10.1111/jvim.15959

**Published:** 2020-11-06

**Authors:** Allison Kendall, Erin Keenihan, Zachary T. Kern, Crystal Lindaberry, Adam Birkenheuer, George E Moore, Shelly L. Vaden

**Affiliations:** ^1^ Department of Clinical Sciences College of Veterinary Medicine, North Carolina State University Raleigh North Carolina USA; ^2^ Department of Molecular Biomedical Sciences College of Veterinary Medicine, North Carolina State University Raleigh North Carolina USA; ^3^ Department of Veterinary Administration College of Veterinary Medicine, Purdue University West Lafayette Indiana USA

**Keywords:** bladder volume, dogs, ultrasound, urine

## Abstract

**Background:**

Although point‐of‐care volumetric assessments of the urinary bladder are not routinely performed in dogs, urine volume quantification can provide important clinical information including noninvasive urine output estimation.

**Hypothesis/Objective:**

Use of 3‐dimensional (3D) ultrasound for determination of urinary bladder volume (UBV) in dogs will be accurate for different bladder volumes and will decrease the need for operator skill in measuring UBV compared to 2‐dimensional (2D) ultrasound evaluation.

**Animals:**

Ten laboratory‐bred Beagle dogs.

**Methods:**

Prospective, experimental study. Urinary bladders were infused with a calculated amount of sterile saline to represent small, medium, and large volumes. Each UBV was estimated and calculated by a board‐certified veterinary radiologist using 3 different 2D ultrasound formulas followed by use of a 3D ultrasound device by a novice. Measured UBVs were compared to the instilled UBV for both 2D and 3D ultrasound methods. Time from start to end of examination was recorded for both ultrasound methods in a subset of dogs.

**Results:**

The 3D ultrasound device underestimated UBV with a mean difference of −9.8 mL compared with 2D ultrasound that overestimated UBV with a difference of +4.2 to 20.3 mL dependent on the 2D formula used. The 3D ultrasound method took less time to measure UBV (mean of 80 seconds per measurement) compared to the 2D method (165 seconds per measurement; *P* = .02).

**Conclusions and Clinical Importance:**

The tested 3D ultrasound device was found to be an accurate and rapid point‐of‐care tool for measuring UBV in dogs, providing a noninvasive method to estimate bladder volume in real time.

**Abbreviations**
2D: 2‐dimensional
3D: 3‐dimensional
AKI: acute kidney injury
H: height
L: length
NCSU‐VH: North Carolina State University Veterinary Hospital
UBV: urinary bladder volume
UOP: urine output
URV: urine residual volume
UTI: urinary tract infection
W: width

## 1 INTRODUCTION

Urinary bladder volume (UBV) and urine residual volume (URV) can provide important clinical information for hospitalized dogs and cats with micturition disorders. Examples of clinical utility include guiding IV fluid therapy in patients with acute kidney injury (AKI), monitoring changes in urine output (UOP) in AKI, diagnosing micturition disorders such as detrusor atony characterized by an abnormally high URV (previously reported median of 0.2 mL/kg),^1^ and avoiding repeated urethral catheterization and catheter‐associated infections.

Urine output can be measured directly by placement of a closed urinary collection system, urethral catheterization, or voided urine collection and measurement.^2^ However, these techniques impose risks such as inaccuracy depending on volume of urine voided, and catheter‐associated urinary tract infections (UTI).^3^, ^4^, ^5^, ^6^ In people, the daily risk of bacteriuria with an indwelling urinary catheter is 3% to 10%,^7^, ^8^ and incidence of catheter‐associated UTI among hospitalized dogs has been reported to be 10% to 19%.^6^, ^9^, ^10^, ^11^ These observations highlight the need for alternative methods to measure UBV other than urinary catheterization.

In dogs, formulations derived from 2‐dimensional (2D) ultrasound measurements have provided acceptable estimates of UBV and for calculating URV to assess UOP.^1^, ^8^, ^9^, ^10^, ^11^, ^12^, ^13^ Formulas derived from the human medical literature have inconsistencies associated with variations in bladder shape and size.^2^, ^12^, ^13^, ^14^, ^15^ Most recently, use of a simple formula of length (L) × width (W) × height (H) × 0.52 has provided the best estimation of bladder volume in people using 2D ultrasound B‐mode images compared to other formulas.^16^ Possible disadvantages of 2D ultrasound include extended duration of time to acquire measurements and postimage calculations, which can be time consuming and may increase the risk of calculation errors. Use of 2D ultrasound and a novel formula in dogs and cats also resulted in marked bias at large urinary volumes (≥264 mL) likely because of the cranial and caudal aspects of the urinary bladder not being visible in sagittal planes at these volumes and at small urinary volumes (≤16 mL).^2^

A 3D‐ultrasound device is used for point‐of‐care volumetric assessment of the urinary bladder in humans as the method of choice for estimating UBV.^17^, ^18^, ^19^, ^20^, ^21^ The use of 3D ultrasound is intended to decrease necessary expertise and training, improve bedside measurement efficiency, and limit examination time.^22^ The use of 3D ultrasound previously has been shown to be more accurate than 2D ultrasonographic methods for quantifying bladder volume in people,^17^ and has been validated recently in dogs.^23^

Our purposes were to (a) assess the validity of 3D ultrasound at variable bladder volumes in dogs and (b) evaluate estimates of 3D bladder volume obtained by a novice and traditional 2D measurements obtained by a board‐certified veterinary radiologist.

We hypothesized that 3D bladder ultrasound for determination of UBV in dogs would be accurate at different bladder volumes, decrease the need for operator skill and result in minimal differences in measured volume compared with 2D ultrasound estimation of bladder volume.

## 2 MATERIALS AND METHODS

### 2.1 Study design and populations

This prospective, experimental study was performed at the North Carolina State University Veterinary Hospital (NCSU‐VH). Mature Beagle dogs from the Laboratory Animal Resources facility at NCSU‐VH were used. Dogs were included if they were clinically healthy as determined by physical examination, CBC and serum biochemistry profile (Antech Diagnostics, Fountain Valley, California) and had no clinical signs of urinary tract disease or history of abnormal urinations.

The study protocol was reviewed and approved by, and conducted in accordance with, the North Carolina State Animal Care and Use Committee.

### 2.2 Data collection

Five intact female Beagle dogs were placed under general anesthesia in compliance with another study protocol (IACUC Protocol Number 19‐066‐O). Dogs received acepromazine (Acepromazine Maleate, Boehringer Ingelheim Vetmedica, Inc, St. Joseph, Missouri) 0.03 to 0.05 mg/kg IV as a premedication before induction. General anesthesia was induced using propofol (Propoflo, Abbott Laboratories Inc, Animal Health Division, North Chicago, Illinois) 3 to 8 mg/kg IV to effect and then maintained by isoflurane (Isoflurane, USP, Halocarbon Products Corporation, North Augusta, South Carolina) 1% to 5% inhalation for the duration of the procedure. Just before bladder scanning, all female dogs received morphine (PF Morphine Sulfate, USP, Baxter Healthcare Corporation, Deerfield, Illinois) 0.1 mg/kg by lumbar epidural route for the purpose of the other study. The dogs were placed in left lateral recumbency and the vulvar region aseptically cleaned using sterile saline and povidone iodine. An 8 Fr indwelling MILA Foley (Female Canine Foley, MILA International, Inc, Florence, Kentucky) urinary catheter then was placed using sterile technique. After urinary catheter placement, the dogs were placed in dorsal recumbency for most accurate 2D ultrasound image acquisition.^13^

Additionally, 5 intact male Beagle dogs were utilized for the purpose of our study alone. Therefore, general anesthesia, as used in the female dogs, was not warranted. Male dogs were sedated using alfaxalone (Alfaxan, Jurox Inc, Kansas City, Missouri) 1 to 3 mg/kg IM or IV with or without the addition of butorphanol (Torbugesic, Zoetis, Kalamazoo, Michigan) 0.2 to 0.3 mg/kg IV depending on the depth of sedation achieved with alfaxalone. They were placed in lateral recumbency and the prepuce and penis were aseptically cleaned using sterile saline and povidone iodine. An 8 Fr red rubber urinary catheter (Dover Red Rubber Catheter, Medtronic [Covidien], Minneapolis, Minnesota) was placed using sterile technique and the dogs were placed in dorsal recumbency for standardization with the female dogs and according to recommendations for 2D ultrasound evaluation.^13^

In all dogs, the urinary bladder was emptied after urinary catheter placement and complete bladder emptying was verified based on 2D‐ ultrasound evaluation (Toshiba Aplio 500, Canon Medical Systems USA, Inc, Tustin, California) performed by a board‐certified veterinary radiologist. The bladder was filled sequentially with a calculated amount of sterile saline (0.9% NaCl, Baxter Healthcare Corporation, Round Lake, Illinois) to represent small (5 mL/kg), medium (7.5 mL/kg), and large (10 mL/kg) bladder volumes. Bladder volume aliquots were determined based on the size of dog and bladder filling without overdistension. These volumes were masked to the observers. Three calculated volumes of sterile saline were utilized for each dog. At any time, if the bladder became distended or turgid, the volume of sterile saline instilled was decreased to a safe maximum volume.

**FIGURE 1:**
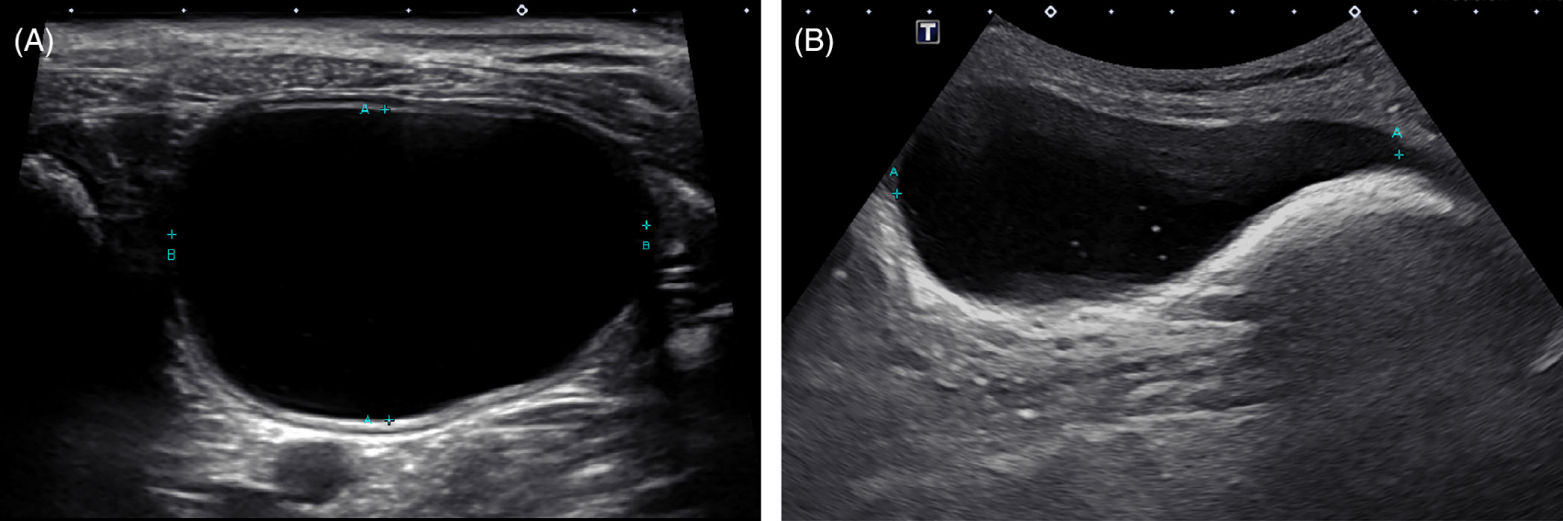
Two‐dimensional ultrasound images depicting width (“B” calipers) and depth (“A” calipers) measurements in transverse (A) and length (“A” calipers) measurement in longitudinal (B). The reader should note that the length measurement used in this study extended into the trigone of the bladder^24^

After each volume of sterile saline was instilled, urinary bladder length, width, and depth were measured by a board‐certified veterinary radiologist using 2D ultrasound (Figure 1). For maximum volume estimation, maximum bladder length on sagittal view was calculated from the bladder apex to the bladder neck.^24^ Each of the 3 measurements was obtained 3 times and the average used to determine 3 calculated volumes.

Because of absence of a standardized linear ultrasonographic measurement in dogs, calculations of bladder volume were performed using 3 different formulas previously reported. The formula L × W × H × 0.52 was used to calculate UBV based on a previously published study in people that reported high accuracy.^16^ The formulas L × W × H × 0.2π and L × W × ([DL + DT]/2) × 0.625 (DL is depth on longitudinal ultrasound; DT is depth on transverse ultrasound) also were calculated based on previous veterinary studies.^2^, ^15^

**FIGURE 2:**
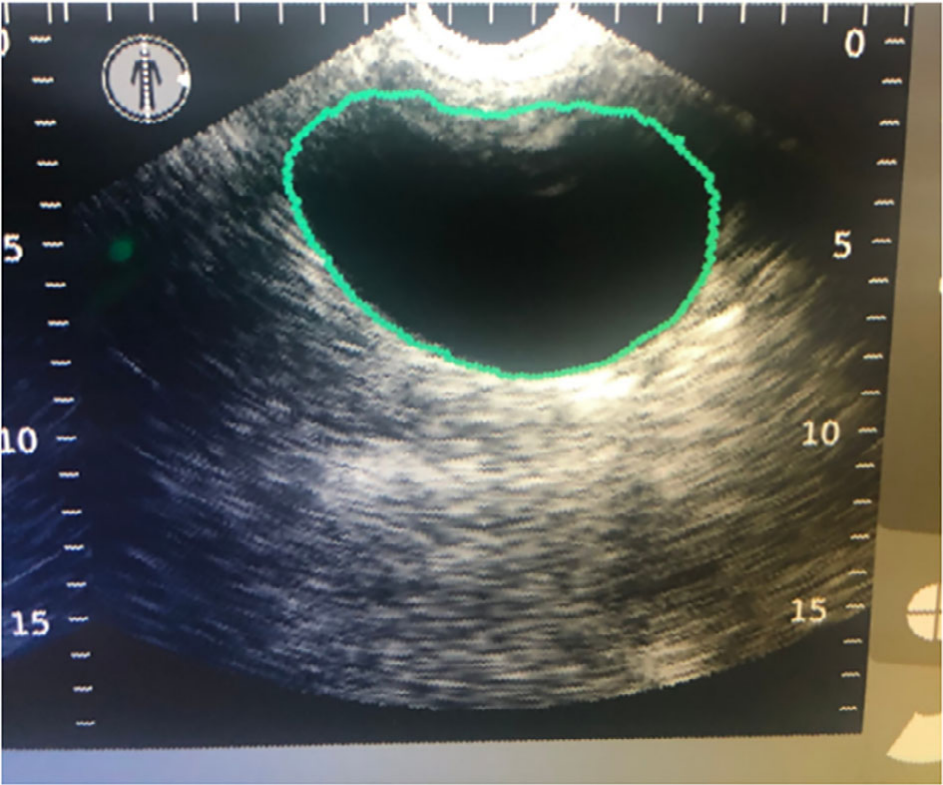
Three‐dimensional Verathon BladderScan Prime Plus depicting measurement of the urinary bladder. The green line represents an “acceptable” measurement of the urinary bladder volume (UBV)

The specified bladder volume subsequently was estimated using the 3D Verathon BladderScan Prime Plus by small animal internal medicine residents according to manufacturer instructions. For the purpose of the study, dogs were placed in dorsal recumbency for comparison of both imaging modalities. The Verathon BladderScan Prime Plus (BladderScan Prime Plus, Verathon, Bothell, Washington) ultrasound probe was placed over the area of the bladder using minimal pressure on the skin. BladderTraq Aiming Assist provided visual clues (a green line was displayed around the image of the bladder) to indicate proper aiming (Figure 2). Immediately after the bladder was in the center of the screen with a surrounding green line, a simple “point and click” technique was used. Within 5 seconds, V_MODE_ technology automatically captured 12 B‐mode slices of the bladder and displayed the calculated volume results on the screen in real time. Three acceptable (green line) measurements were acquired for each instilled volume, and subsequently averaged to determine final UBV.

After completing examinations in the first 2 male dogs, it seemed likely that the 3D ultrasound method was faster than 2D ultrasound. At this point, we added assessment of time as an additional study objective. The time from start to end of examination was recorded for both 2D (not including calculations) and 3D ultrasound measurements in 3 of the 5 male dogs, and none of the female dogs. Time was defined as the point at which the ultrasound probe touched the skin to the point the ultrasound probe was removed from the skin.

### 2.3 Statistical analysis

Data were analyzed using commercial software (MedCalc Software Ltd, version 19.2.5, Ostend, Belgium). Numerical data were assessed for normality using the Shapiro‐Wilk test. Because of the exploratory nature of the study and small sample sizes, descriptive rather than inferential statistics were utilized to eliminate type II statistical errors. Variables were described as mean ± SD. Ranges also are reported because of small sample size. A scatter plot was constructed for comparison of UBV measurements by the 2 ultrasound devices.

Bland‐Altman analyses were performed for the 3 different 2D ultrasonographic formulas for estimating UBV. An unpaired *t* test was used to compare time between the 2 ultrasound devices. Statistical significance was set at *P* < .05.

## 3 RESULTS

Ten dogs (5 males and 5 females) were evaluated (Table 1). Mean ± SD body weight was 10.9 ± 1.2 kg with a range of 8.7 to 12.8 kg.

Four of the 10 dogs were unable to tolerate the maximum volume aliquots without overdistension noted by abdominal palpation and bladder wall stretching observed by 2D ultrasound. These dogs had their volume aliquots decreased to a safe maximum volume.

### 3.1 Results of 2D B‐mode ultrasound formulas for estimation of UBV

**FIGURE 3:**
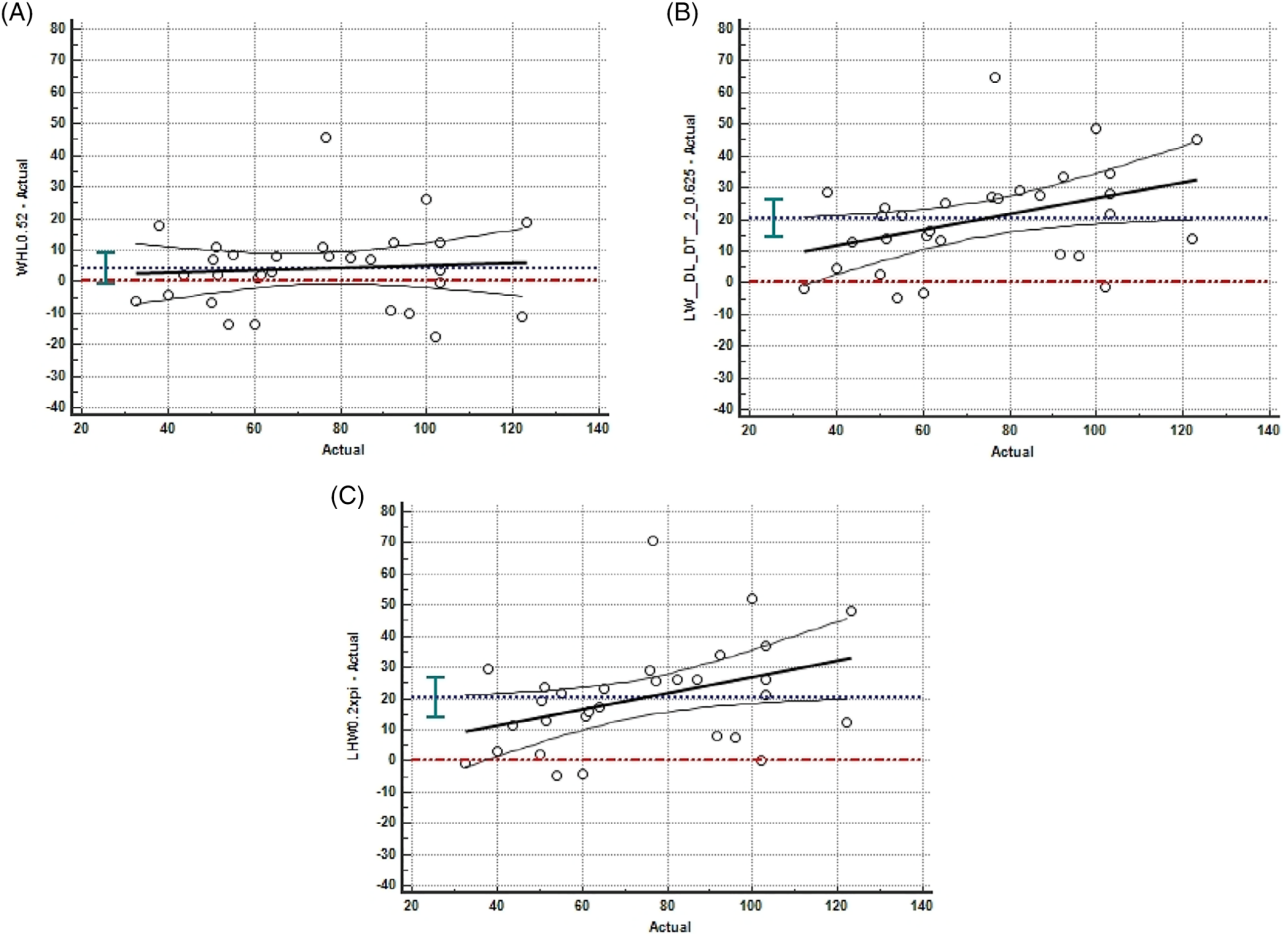
Bland‐Altman plots for A, formula 1 (L × W × H × 0.52); B, formula 2 (L × W × ([DL + DT]/2) × 0.625); and C, formula 3 (L × W × H × 0.2π). The solid black line represents the line of regression (proportional bias) and the blue dashed lines represent the overall mean difference between calculated and actual instilled urinary bladder volume (UBV)

Calculation of UBV using the formula L × W × H × 0.52 (formula 1) compared to the known instilled volume resulted in a mean difference of 4.2 ± 13.1 mL. Calculation of UBV using the formula L × W × ([DL + DT]/2) × 0.625 (formula 2) compared to the known instilled volume resulted in a mean difference of 20.2 ± 15.9 mL. Calculation of UBV using the formula L × W × H × 0.2π (formula 3) compared to the known instilled volume resulted in a mean difference of 20.3 ± 17.1 mL. Bland‐Altman graphs for comparison among the 3 formulas are presented in Figure 3. The length measurement used extended into the trigone of the bladder, which may not have reflected the methodology of other published 2D formulas, and thus may have adversely affected 2D‐calculated UBV (Figure 1).

No significant difference was found between estimated UBV and actual instilled UBV using formula 1 (*P* = .67). In analysis of estimated UBV using the other 2 reported formulas compared to actual UBV, both mean difference in volume and slope of the regression lines differed significantly from 0 with formula 2 (*P* = .03) and formula 3 (*P* = .03). Based on these results, formula 1 (L × W × H × 0.52) was used to compare accuracy between the estimated UBV of 2D and 3D ultrasound against the known instilled volume.

### 3.2 Results of 2D ultrasound and 3D BladderScan ultrasound

**FIGURE 4:**
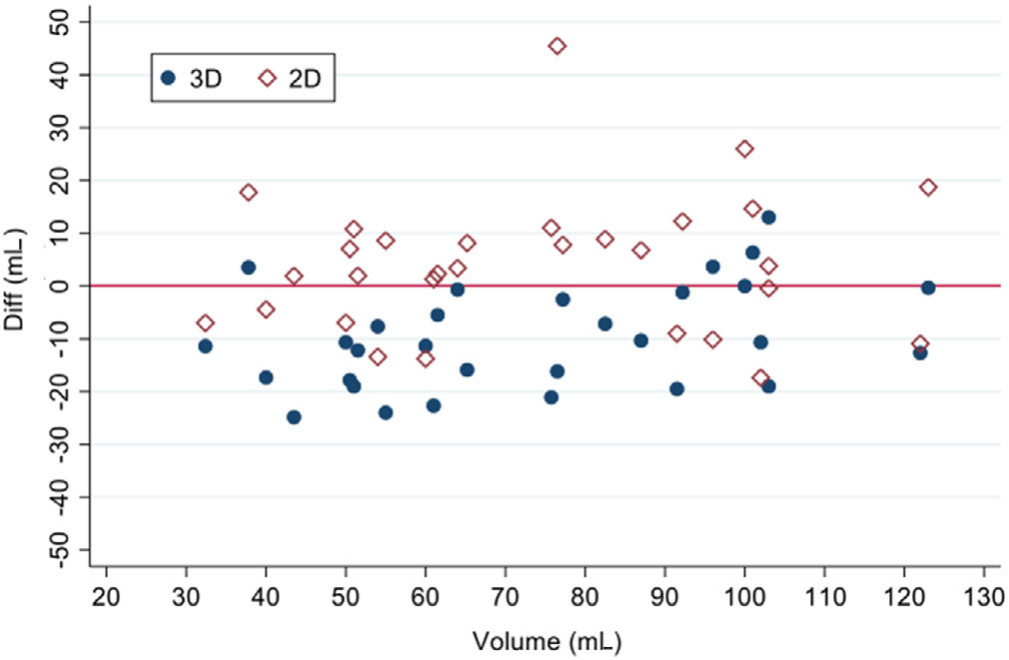
Scatter plot of difference in infused volume minus ultrasound‐calculated urinary bladder volume for both 2D (open diamonds) and 3D (closed circles) compared to infused volume. Two‐dimensional ultrasound formula depicted is formula 1 (L × W × H × 0.52)

Calculation of UBV using the most accurate 2D ultrasound formula^14^ (formula 1) compared to the known instilled volume resulted in a mean difference of 4.2 ± 13.1 mL and an observed range of −17.4 to 45.5 mL (62.9 mL). Thus, the 2D ultrasound formula tended to overestimate UBV at different bladder volumes (Figure 4).

Estimation of UBV using 3D ultrasound resulted in a mean difference of −9.8 ± 9.8 mL and an observed range of −24.8 to 13.0 mL (37.8 mL). Thus, the 3D ultrasound device tended to underestimate bladder volume (Figure 4).

### 3.3 Time to perform 2D and 3D ultrasound measurements

The time from start to end of examination was recorded for both 2D (not including calculations) and 3D ultrasound measurements in 3 of the 5 male dogs. Time was defined as the point at which the ultrasound probe touched the skin to the point the ultrasound probe was removed from the skin. The 3D ultrasound method required less time to measure UBV with a mean ± SD of 80 ± 29 seconds (range, 45‐120 seconds) per measurement compared to 165 ± 24 seconds (range, 121‐210 seconds) per measurement (not including calculations) for 2D ultrasound (*P* = .02).

## 4 DISCUSSION

We determined that use of a 3D ultrasound device was safe, efficient, and clinically effective for measuring UBV in dogs. The 3D ultrasound unit is portable and can be used “cage‐side” in hospitalized patients similar to monitoring UBV in people. The device is especially useful because it avoids urethral catheterization, which eliminates the risk of catheter‐associated UTI and decreases discomfort associated with catheterization.

Overall, the use of a 3D ultrasound device by a nonradiologist tended to underestimate bladder volume compared to 2D ultrasound assessment by a radiologist, which tended to result in overestimation of UBV. This finding is similar to what is observed in humans, in whom 3D ultrasound underestimated actual bladder volume and 2D ultrasound overestimated it.^19^, ^25^ Based on subjective visual evaluation in our study, underestimation by 3D ultrasound was greater at small bladder volumes. This observation is in contrast to a study in humans that found both 2D and 3D ultrasound devices overestimated bladder volume at lower filling (<160 mL) and underestimated volume at higher filling (>160 mL).^26^ The scan underestimation could be explained partly by leakage of saline around the urinary catheters during 2D estimations before 3D measurements, or failure of 3D ultrasound to accurately capture the bladder at small urinary volumes (small urinary volumes in our study ranged from 32.4 to 64 mL, which is considerably smaller than volumes in the studies of humans). The 3D ultrasound device also does not capture the bladder neck and thus could have underestimated volume compared to 2D ultrasound in which the bladder neck was included for maximum length measurement. Ultimately, understanding the extent of variation is important for clinical assessment, and was achieved by our study.

Use of the 3D ultrasound device required less time to estimate UBV than did use of 2D ultrasound. Based on our findings, 3D ultrasound consistently took less than 2 minutes to obtain an average measurement, consisting of 3 to 5 measurements. The scanner produces an average volume (mL) and degree of accuracy in real time on the screen. This would be especially useful in clinical applications when quick, efficient estimation of bladder volume is needed in a hospital setting, without the need for postimage calculations. Examples include monitoring UOP in AKI patients, monitoring for urine retention postoperatively, and diagnosing micturition disorders based on URV. The device also allows for quick estimation “cage‐side” without need for special operator skill, which would be especially useful in hospitalized dogs.

Dogs in our study were anesthetized or sedated for urinary catheter placement and bladder filling, but use of the 3D ultrasound device in the clinic likely would require no sedation and minimal restraint, which would be especially useful for clinically ill dogs. The 3D ultrasound UBV estimation was acceptable in our study, and future studies involving standing, unsedated dogs in a clinical setting are warranted. Although postoperative urinary retention caused by anesthetic and opioid use has been well documented in people and dogs,^27^, ^28^, ^29^, ^30^, ^31^, ^32^ the bladders of the dogs in our study were completely emptied of urine and filled with calculated amounts of sterile saline. Our study did not assess bladder capacity, and thus these effects should not have affected bladder filling.

Our study had several limitations. We were unable to directly compare the accuracy of 3D and 2D ultrasound imaging because they were performed by different operators. However, the purpose of our study was to evaluate 3D ultrasound using a known amount of saline in the bladder, which is useful as a clinical correlate. The second purpose was to determine if 3D ultrasound could be used by a novice with minimal to no ultrasound training and produce results clinically comparable to those obtained by a board‐certified veterinary radiologist using 2D linear and convex ultrasound probes. This objective is important because the goal of using 3D ultrasound device is to allow rapid, “cage‐side” estimations of bladder volume by individuals with minimal training.

Several 2D linear ultrasonographic formulas have been reported in the literature.^1^, ^2^, ^12^, ^13^, ^14^, ^15^, ^33^ However, no consensus has been reached in veterinary medicine about which formula is most accurate. Thus, our study reported a formula described in the human medical literature as being most accurate.^16^ When compared to 2 previously reported formulas from the veterinary medical literature, the formula L × W × H × 0.52 resulted in less discrepancy between actual and infused UBV. Because the purpose of our study was to determine how UBV as measured by 3D ultrasound compared to the gold standard of 2D ultrasound measurements, and not that 3D ultrasound was superior to 2D ultrasound, we elected to use the most accurate 2D linear ultrasonographic measurement. In our study, 3D ultrasound estimated UBV with greater precision than did the 2 formulas for 2D ultrasound previously reported in the veterinary medical literature.

Additional studies should include the same operator utilizing both ultrasound devices to directly compare the accuracy of the 3D ultrasound device to traditional 2D ultrasound measurements.

Because we used dogs from another study, our sample size was small and included a single standard breed and size of dog. However, the bladders were emptied and filled with calculated amounts of sterile saline to represent different bladder volumes. Bladder volume aliquots were determined based on the size of dog and bladder filling without overdistension. Previous studies have suggested 3.5 mL/kg as normal bladder volume and up to 20 mL/kg as maximum bladder volume.^1^, ^34^, ^35^ Initially, 20 mL/kg was chosen to represent the large bladder volume, but we noted overdistension by abdominal palpation and bladder wall stretching on 2D ultrasound at 7.5 to 10 mL/kg in all dogs. We suspect this difference maybe because the dogs in our study lived in a research facility and were not housebroken, which might have contributed to smaller capacity bladders, and thus smaller UBV ranges. Because of our finding of greater underestimation at small volumes, use of larger size dogs with larger capacity bladders might have produced more reliable results and a larger range of bladder sizes. A previous study in dogs found that the accuracy of 3D ultrasound varied between the size of dogs and device setting utilized.^23^ However, in our study we used a newer model of the Verathon BladderScan Prime Plus which automatically adapts to patient size and no longer has separate adult and child settings. Additional studies are warranted to investigate the accuracy of the 3D ultrasound device in different sizes of dogs and to determine if it is clinically useful in cats.

All dogs were scanned in dorsal recumbency because of increased accuracy for 2D ultrasound.^13^ Dogs were in dorsal recumbency for both 2D and 3D ultrasound measurements so as to compare the methodologies in the same body position. An advantage of 3D ultrasound is its ability to automatically adjust to patient size, bladder shape, and the ability to capture the bladder using ImageSense technology independent of body position (according to Verathon manufacturer claims).^17^ This is in contrast to 2D ultrasound where, at large volumes, the cranial and caudal poles of the bladder may not be visible in a single view because of interference of the prepubic bone and abdominal structures.^12^ Additional studies evaluating the 3D ultrasound device using dogs in lateral recumbency or in a standing position and unsedated would be helpful.

The manufacturer recommends approximately 30 minutes to 1 hour of training on the PrimePlus BladderScan before use to ensure accurate results. In our study, the 2 evaluators using 3D ultrasound had minimal prior training utilizing 2D ultrasound but only received 5 to 10 minutes of training before using the 3D scanner with minimal practice on clinical patients beforehand because the scanner was obtained shortly before initiating the study. The previous study in dogs reported that an experienced operator determined a significantly higher mean volume than did novice.^23^ Therefore, we believe more extensive formal training of clinical staff might result in more accurate bladder measurements.

In conclusion, a point‐of‐care 3D ultrasound device may allow rapid, “cage‐side” volumetric assessment of the urinary bladder in dogs and requires little expertise and training. The device could be used for daily monitoring of bladder volume in dogs and serve as a tool for diagnosis of micturition disorders without the need for urethral catheterization and unnecessary risk of catheter‐associated UTIs. Additional studies are warranted to validate accuracy in comparison to traditional 2D ultrasound measurements when conducted by users with the same level of expertise and to evaluate use of the device in clinical scenarios such as monitoring urinary retention and assessing urine production.

## CONFLICT OF INTEREST DECLARATION

Shelly L. Vaden serves as Associate Editor and George E. Moore serves as Consulting Editor for Experimental Design and Statistics for the Journal of Veterinary Internal Medicine. They were not involved in review of this manuscript.

## OFF‐LABEL ANTIMICROBIAL DECLARATION

Authors declare no off‐label use of antimicrobials.

## INSTITUTIONAL ANIMAL CARE AND USE COMMITTEE (IACUC) OR OTHER APPROVAL DECLARATION

Approved by the North Carolina State Animal Care and Use Committee (Protocols 19‐656 and 19‐066‐O).

## HUMAN ETHICS APPROVAL DECLARATION

Authors declare human ethics approval was not needed for this study.

## Supporting information


**Supplemental Table 1** Demographics for the study population including sex, weight and instilled urinary volumes.
