# Supplementary material for: Three‐dimensional bladder ultrasound for estimation of urine volume in dogs compared with traditional 2‐dimensional ultrasound methods
Source: J Vet Intern Med. 2020 Nov 6;34(6):2460–7. doi: 10.1111/jvim.15959 (PMC7694864; doi:10.1111/jvim.15959)
Supplement: Supplementary file 1 — Supplemental Table 1 Demographics for the study population including sex, weight and instilled urinary volumes. [file JVIM-34-2460-s001.pdf]

Supplemental Table 1: Demographics for the study population including sex, weight and instilled urinary volumes.

Patient (sex)	Weight (kg)	Volume (ml/kg)	Volume (ml)
1 (FS)	10.2	5	51
		7.5	76.5
		10	102
2 (FS)	8.7	5	43.5
		7.5	65.2
		10	87
3 (FS)	12.2	5	61
		7.5	91.5
		10	122
4 (FS)	11	5	55
		7.5	82.5
		9	100
5 (FS)	10.3	3.9	40
		4.9	50
		5.8	60
6 (MC)	12.8	5	64
		7.5	96
		8	103
7 (MC)	10.3	5	51.5
		7.5	77.2
		10	103
8 (MC)	10.1	5	50.5
		7.5	75.75
		10	101
9 (MC)	10.8	3	32.4
		3.5	37.8
		5	54
10 (MC)	12.3	5	61.5
		7.5	92.2
		10	123
